# Music to the Ears: An Unusual Case of Frontal Lobe Stroke With Complex Auditory Hallucinations

**DOI:** 10.7759/cureus.31127

**Published:** 2022-11-05

**Authors:** Arielle Degueure, Andee Fontenot, Muhammad W Khan, Ammar Husan

**Affiliations:** 1 Medicine, Louisiana State University Health Sciences Center, Shreveport, USA; 2 Neurology, University of Rochester Medical Center, Rochester, USA

**Keywords:** charles bonnet syndrome, stroke, frontal lobe, auditory cortex, hallucinations

## Abstract

Auditory hallucinations are defined as the perception of sensory auditory input in the absence of an external stimulus. It is a multifaceted pathology with a range of symptoms and an even wider range of possible underlying causes. Its lack of true distinctive clinical features along with overlapping symptoms makes distinguishing between etiologies difficult without appropriate workup. Here, we present an unusual case of left frontal lobe ischemic infarction, resulting in complex musical auditory hallucinations in the absence of behavioral changes.

## Introduction

Auditory hallucinations (AH) can range from simple nonspecific sounds in the form of tinnitus, to complex manifestations involving voices and music [1,2]. Characteristic features described by patients may shine a light on potential underlying causes of AH. Psychotic spectrum disorders, such as schizophrenia, more commonly present with auditory hallucinations that are verbal in nature [3,4]. Often referred to as “command” hallucinations with voices speaking in the second or third person. In contrast, musical forms of hallucinations, specifically consisting of church choral music are said to be typical of neurodegenerative disorders and auditory Charles Bonnet Syndrome, which will be discussed later [4].

Musical hallucinations (MH) represent a rare and complex subtype of auditory hallucination characterized by a false perception of melodies, songs, and music [5]. Musical hallucinations are often familiar to the patient; however, this is not always the case [3]. An in-depth understanding of etiologies resulting in AH has not quite been achieved. However, various conditions, such as hearing impairment, psychiatric disorders, focal brain lesions, generalized brain atrophy, epilepsy, and intoxications, are suggested as predisposing factors for the development of MH [2].

Various neurotransmitters have been described relating to the underlying neurobiological mechanism of MH. Choline, gamma-aminobutyric acid (GABA), serotonin, and dopamine have all been described in relation to their modulatory effects on MH symptoms [6]. Their physiologic effects and pharmacological implications will be discussed further in this report in addition to highlighting the complexity of MH and gaps in our understanding of the current pathophysiology driving the need for further research.

## Case presentation

Appropriate protocols were followed, and consent was obtained. This project was approved by the hospital's institutional review board. A 97-year-old woman with a history of presbycusis presented with dysarthria and a right-sided facial droop, suggesting a possible left-sided cerebrovascular accident (CVA). These symptoms were preceded by new intermittent auditory hallucinations onset 24-36 hours prior. During that time, her condition progressed with worsening of AH in addition to the development of dysarthria and right-sided facial droop, prompting her to seek medical evaluation. Auditory hallucinations were musical in nature and familiar to the patient. She reported hearing "Ave Maria" with clear and completely formed choral lyrics and accompanying instrumental music. Each episode lasted several minutes, followed by a complete resolution. The patient was not fearful or in distress over her hallucinations and retained insight regarding their false nature. Neither the patient nor her close contacts endorsed any behavioral or psychiatric changes during this time or the period leading up to her symptoms. No prior neurologic history was reported, aside from age-related hearing loss without tinnitus or the use of hearing aids. The patient was able to carry out daily activities of living and managed her own finances and groceries. No acute neurocognitive decline or psychiatric changes were reported.

**Figure 1:**
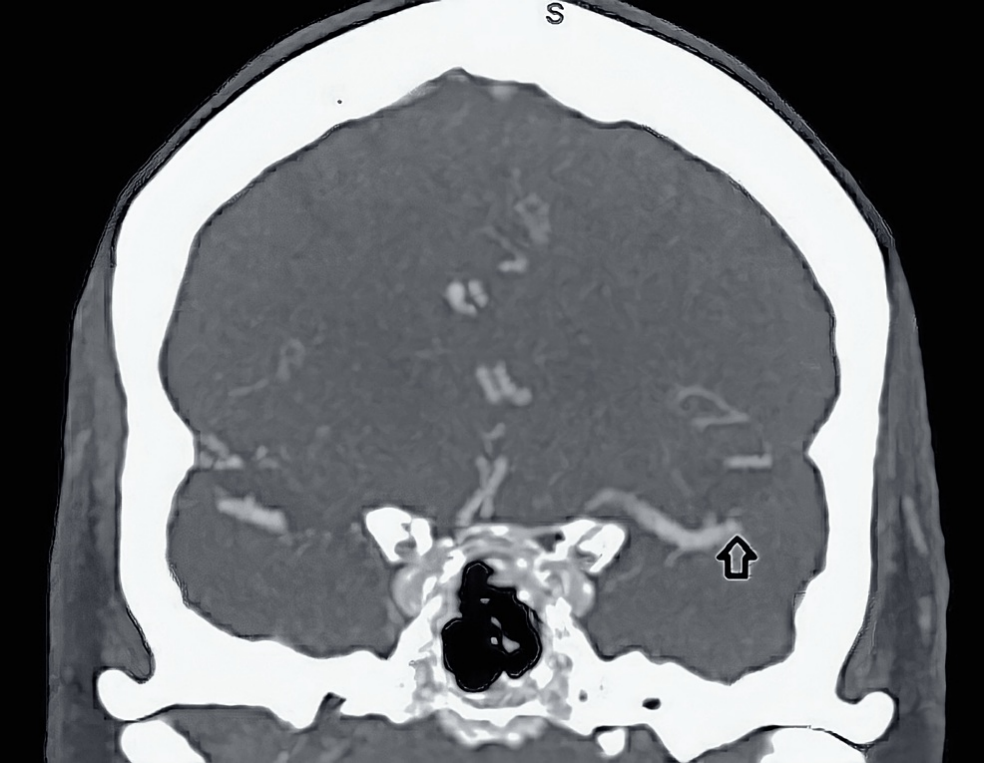
Patient's computed tomography angiogram (CTA) CTA imaging demonstrated stenosis of the M2 segment of the left middle cerebral artery.

**Figure 2:**
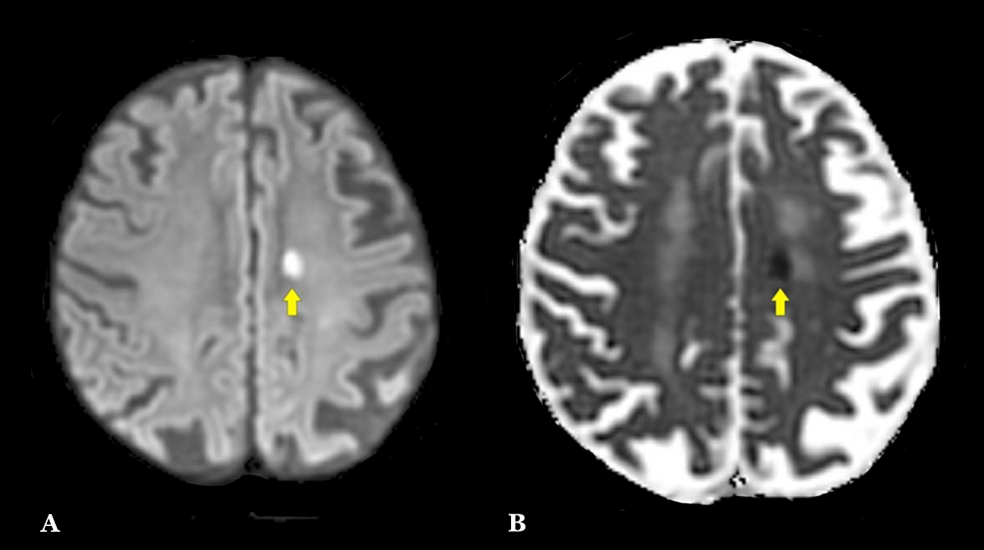
Patient’s magnetic resonance imaging (MRI) sequences The diffusion-weighted imaging (DWI) sequence in Panel A shows subcortical restricted diffusion along the left-sided periventricular region of the parietal lobe. Panel B shows the apparent diffusion coefficient (ADC) correlate of the same region signifying cytogenic edema due to acute/subacute ischemic infarction.

**Figure 3:**
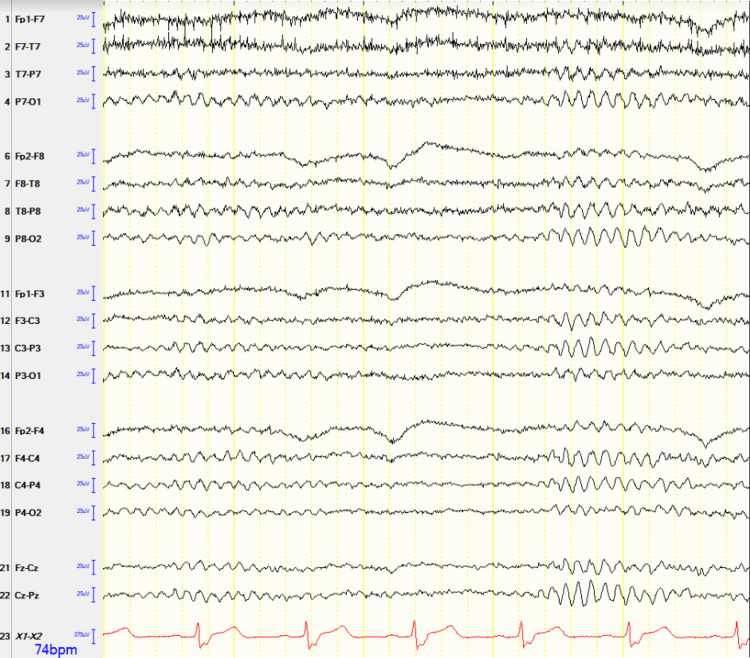
Patient’s double montage electroencephalograms Normal bipolar montage EEG with no electrographic abnormalities suggestive of seizures.

On arrival, the patient was hemodynamically stable, and computed tomography (CT) without contrast and computed tomography angiogram (CTA) were performed with no signs of hemorrhage (Figure 1). However, stenosis of the left-sided M2 branch of the middle cerebral artery (MCA) was reported as the likely underlying etiology. Additionally, magnetic resonance imaging (MRI) of the brain revealed left frontal subcortical and periventricular ischemic infarcts (Figure 2). The further AH investigation prompted continuous 48-hour electroencephalogram (EEG) monitoring, which failed to reveal any epileptiform or seizure-like activity despite persistent AH throughout the monitoring (Figure 3). Metabolic and toxicology workups were performed, yielding values within the normal range (Table 1). The infectious workup was significant for nitrites and leukocytes on urine analysis (Table 2), suggestive of a urinary tract infection that was treated with ceftriaxone. Despite treatment and resolution, musical auditory hallucinations persisted throughout her hospital course, suggesting symptoms independent of her urinary tract infection.

**Table 1:** Serum laboratory tests and urine drugs screen results

Laboratory Test	Result
Complete blood count	Within normal limits
Complete metabolic panel	Within normal limits
Blood glucose	136 mg/dL
Blood culture	Negative for growth
Urine drug screen	Negative

**Table 2:** Urine analysis and results

Urine Analysis	Results	Reference
Color	Yellow	Yellow, Straw, Amber
Specific gravity	1.020	​​1.005-1.030
Leukocytes	2+	Negative
Nitrites	Positive	Negative

The patient’s ischemia was managed following standard practice guidelines, including aspirin, atorvastatin, and clopidogrel. After clearance from the speech-language pathology department in regard to her dysarthria and facial paralysis, the patient was discharged on the appropriate medications. At this time, the patient continued to report musical auditory hallucinations after all other presenting symptoms had resolved. Follow-up visit two months later, improvement of MH was reported but failed to reach a complete resolution.

## Discussion

As described earlier, musical hallucinations (MH) are complex AH characterized by the false perception of songs, melodies, or rhythms in the absence of music [5]. MH is an uncommon form of AH whose prevalence has not yet been accurately reported. The largest and most recent review of 393 cases of MH identified five categories of associated comorbid conditions: neurological, psychiatric, structural, drug effect, and not otherwise classifiable [4]. Some studies report MH in 0.16% of general psychiatric admissions and up to 2.5% of elderly patients with hearing impairment or other neurodegenerative disorders [3,5,7]. Over the years researchers have continued to study the prevalence of MH in order to better understand its epidemiology.

The neurobiological mechanism underlying MH is not yet fully understood. However, various neurotransmitters are reported to influence the severity of MH symptoms. A choline deficiency is thought to play a role in MH, in part due to a worsening of symptoms reported with anticholinergic drug use [6,8]. Dopamine is thought to be responsible for the pleasure or familiarity associated with MH [8]. High dopamine levels in the limbic system are described in the setting of hallucinations reported in cases of schizophrenia [9]. In contrast, with age, a decrease in GABA released in the auditory nuclei is said to result in symptoms of hypoacusis. Cases of MH associated with hearing impairment were also found to have symptom improvement with the administration of gabapentin [6]. Ultimately, cases studies of MH caused by etiologies such as hypoacusis and neurocognitive and psychiatric disorders suggest that modulation of neurotransmitters via gabapentin, anticholinergic, and antipsychotic drugs play role in the altering severity of symptoms [6,8]. However, not all patients with a history of hypoacusis and anticholinergic drug use develop MH, suggesting that the development of MH is more complex than a neurotransmitter imbalance alone.

Early cases of MH analyzed by Berrios and Keshavan et al. found an average age of 60 years with an 80% female predominance [10,11]. Isolated MH was reported in only 40% of cases, suggesting most MH presented with additional neurologic symptoms [12]. Interestingly, the most common association, accounting for 67% of cases was hearing loss [10,13]. This association is thought to be due to an adaptive reduction in sensory precision as a result of hearing loss, resulting in inferred hallucinatory perceptions [13]. The deafferentation phenomenon is also described in the context of visual hallucinations secondary to vision loss [14]. Lack of sensory input leads to a phantom sensory perception, manifesting as what is known as Charles Bonnet Syndrome [12]. In contrast to overall impairment, MH in cases with asymmetric hearing impairment is thought to be due to incomplete suppression or sensory input of the contralateral ear [7]. Neuroimaging studies of musical AH in cases of acquired deafness (without infarction) suggest cortical involvement via complex networks involving both the temporal and frontal cortex [15]. Applying this network-based mechanism to these same areas in the context of infarction could explain a clinical presentation with overlapping features of both temporal and frontal cortex involvement.

In our patient, stenosis of the M2 branch of the left MCA along with frontal subcortical and periventricular infarcts led to MH. The anatomy of the M2 segment can be further subdivided into superior and inferior divisions [16]. The superior division supplies the lateral inferior frontal lobe, which on the dominant (usually left) hemisphere houses Broca's area [16]. Pathologies of this region often present as dysarthria, as in the case of our patient. The inferior division of the M2 segment supplies the lateral superior temporal lobe, which includes the auditory cortex in Heschl's gyrus and in the case of the dominant hemisphere, Wernicke's area [16]. Involvement of the superior temporal sulcus has been suggested in MH due to its observed activation in response to an external auditory stimulus [17]. However, PET imaging during active musical hallucinations showed activation of the posterior temporal lobes and inferior frontal cortices in addition to the basal ganglia and cerebellum without activation of the primary auditory cortex [18].

Based on our review of prior reports, MH secondary to M2 segment pathology is, in fact, possible [17,18]. However, the conflicting symptoms or lack thereof in our patient make this case unique. Cases of insular infarcts involving the M2 segment of the MCA territory consistently report psychiatric or behavioral changes in conjunction with hallucinations [19]. These findings are likely due to the shared MCA blood supply to both the frontal lobe and the temporal lobe. Though the pathophysiology is not completely understood, it has been hypothesized that the frontal lobe plays a role in activating hallucinatory pathways by altering the level of awareness and stimulating internal speech, illustrating frontal-lobe infarcts contributing to the emergence of complex hallucinations [17].

Our patient failed to display behavioral changes but experienced other characteristic features of MCA pathology involving the dominant hemisphere such as dysarthria [16]. MH originating from this region in the absence of psychiatric changes has been observed in a case of an insular glioma located between the M2 and M3 segments [20]. However, this was a right-sided pathology involving the non-dominant hemisphere. Early case reviews have suggested that right-sided, or non-dominant, hemisphere pathology is more significant in the development of MH [10,12]. However, analysis by Keshavan et al found that in cases secondary to coarse brain disease, right-sided lesions are only marginally more often associated with MH [11,12]. Further comparison between right and left-sided lesions in the setting of MH is needed to establish whether or not lateralization is significant in MH development.

## Conclusions

Here, we presented an unusual case of a 97-year-old woman with musical auditory hallucinations secondary to acute left frontal subcortical and periventricular ischemic infarcts in the absence of personality or behavioral changes. Strokes involving the insular cortex, specifically regions supplied by the M2 MCA segment, typically present with personality changes with possible psychiatric features. Furthermore, MH has historically been more commonly reported in right-sided lesions. Our patient’s left-sided pathology and lack of coinciding psychosis make this case interesting. To our knowledge, this is the first report of complex musical hallucinations involving the dominant hemisphere without associated behavioral symptoms. This case provides little evidence for any significant neurotransmitter imbalance outside of neurovascular injury other than a history of hearing loss. However, no treatment with gabapentin was administered in order to confirm whether low levels of GABA contributed to symptoms. This case demonstrates the complexity of MH and suggests that isolated lesions or neurotransmitter imbalances are likely underestimating the complexity of downstream effects. Further research into the neurobiological cause of musical hallucinations is needed to better grasp the mechanism of this pathology and how it relates to our patient’s unique presentation.
